# Towards personalized agriculture: what chemical genomics can bring to plant biotechnology

**DOI:** 10.3389/fpls.2014.00344

**Published:** 2014-07-11

**Authors:** Michael E. Stokes, Peter McCourt

**Affiliations:** Department of Cell & Systems Biology, University of TorontoToronto, ON, Canada

**Keywords:** herbicides, chemical genetics, agricultural biotechnology, growth regulators, chemical screening, genomics

## Abstract

In contrast to the dominant drug paradigm in which compounds were developed to “fit all,” new models focused around personalized medicine are appearing in which treatments are developed and customized for individual patients. The agricultural biotechnology industry (Ag-biotech) should also think about these new personalized models. For example, most common herbicides are generic in action, which led to the development of genetically modified crops to add specificity. The ease and accessibility of modern genomic analysis, when wedded to accessible large chemical space, should facilitate the discovery of chemicals that are more selective in their utility. Is it possible to develop species-selective herbicides and growth regulators? More generally put, is plant research at a stage where chemicals can be developed that streamline plant development and growth to various environments? We believe the advent of chemical genomics now opens up these and other opportunities to “personalize” agriculture. Furthermore, chemical genomics does not necessarily require genetically tractable plant models, which in principle should allow quick translation to practical applications. For this to happen, however, will require collaboration between the Ag-biotech industry and academic labs for early stage research and development, a situation that has proven very fruitful for Big Pharma.

## INTRODUCTION

Historically, the pharmaceutical industry has developed drug treatments that target the widest segment of the population. Although this business model has been very successful, there is a need to update this “one size fits all” approach to drug development. Genetic variability in the human population renders some individuals less responsive to certain therapies (McDonald et al., 2009). More importantly, an individuals’ genetic makeup can make them susceptible to dangerous side effects from the medication (Daly et al., 2009). This has led to suggestions that drug treatments need to take into account a patients’ genome, hence the development of the field of pharmacogenomics (Weinshilboum and Wang, 2006; Wang et al., 2011). By tailoring drugs regimens to the needs of the individual based on their unique set of alleles, more effective and safer therapies can be prescribed (Ginsburg and Willard, 2009).

Understanding the molecular basis of disease is fundamental to designing selective drug treatments. For example, over 1500 different mutations in the cystic fibrosis transmembrane conductance regulator (CFTR) gene have been identified in cystic fibrosis (CF) patients^1^. Although 90% of CF patients have an in-frame deletion that results in the mislocalization of the CFTR gene product, a small fraction of CF patients (~5%) have a missense mutation G551D-CFTR that has correct CFTR localization but reduced chloride channel activity (Van Goor et al., 2011). Using this allelic information, researchers identified compounds that specifically rectify the perturbation caused by each CFTR allele (Ramsey et al., 2011; Van Goor et al., 2011). For example, the drug Ivacaftor binds the ion channel to promote chloride transport in patients harboring the G551D-CFTR allele (Yu et al., 2012; McPhail and Clancy, 2013). Ivacaftor has been developed into a clinically effective therapeutic under the trade name Kalydeco (Whiting et al., 2014). During the development of novel CF therapeutics, genetics informed the drug discovery process and enabled high-throughput screening to identify compounds that selectively targeted each allele.

The parallels between the pharmaceutical and agricultural chemical industry are striking. As with many pharmaceuticals, the foundation of the Ag-chemical industry is the identification of chemicals that have generalized benefits to a wide variety of crops. Popular herbicides kill plants by targeting vital processes conserved across plant biology but not found in mammals, such as photosynthesis or amino acid biosynthesis (**Table 1**; Shaner, 2004), however, a broad-spectrum herbicide that targets a common process in plants may not prove beneficial to a farmer that is trying to selectively kill one type of plant while preserving another. To overcome this issue, inventive Ag-biotech companies deal with the indiscriminate action of these compounds by engineering transgenic crops (GMOs) for herbicide resistance (Mazur and Falco, 1989; Funke et al., 2006; Pollegioni et al., 2011). This approach worked famously well for Monsanto in the development of *Roundup Ready* crops that have been engineered for resistance to glyphosate, the active ingredient in the herbicide *Roundup* (Padgette et al., 1995). Glyphosate binds and inhibits the 5-enolpyruvylshikimate-3-phosphate (EPSP) synthase enzyme, the penultimate step in the shikimate biosynthesis (Padgette et al., 1995; Funke et al., 2006). *Roundup Ready* plants express a microbial EPSP synthase that does not bind glyphosate, and are therefore resistant to the inhibitory effect of the herbicide (Padgette et al., 1995; Funke et al., 2006). In this way, spraying herbicides over engineered crops enables farmers to inhibit all plant growth aside from the desired resistant plants (Padgette et al., 1995).

**Table 1:** Herbicide mode-of-action and chemical targets.

Mode of action	Site of action	Chemical family	Resistant weed species (U.S.)
Lipid synthesis	Acetyl CoA carboxylase (ACCase)	Arloxyphenoxy propionate	15
		Cyclohexanedion	
Amino acid synthesis	Acetolactate synthease (ALS)	Sulfonylurea	38
	5-enolpyruvyl-shikimate-3-phosphate	Glycine	7
	synthase (EPSP)		
Growth regulators	Auxin receptor	Phenoxy-carboxylic acid	7
	Auxin transport	Benzoic acid Semicarbazone	
Photosynthesis	Photosystem II electron transport	Triazine, trazinone,	22
		Nitrile	1
		Benzothiadiazole,	7
		Ureas	
	Photosystem 1 electron transport	Bipyridilium	
Nitrogen metabolism	Glutamine synthase	Phosphonic acid	0
Pigment inhibitors	Diterpene synthase	Isoxazolidinone	0
	Hydroxyphenylpyruvate	Isoxazole, triketone	0
	dioxygenase		
Cell membrane disruptor	PPO inhibitors	Diethylether,	2
		*N*-phenylphthalimide,	4
		Thiadiazole	
Seedling root growth	Microtubule inhibitors	Dinitroaniline	6
Seedling shoot growth	Lipid synthesis	Thiocarbamate	5
	(non-ACCase)		
	Long chain fatty acid inhibit	Chloroacetamide	1

*Broad-spectrum herbicides target a wide range of plant-specific processes, including photosynthesis and amino acid biosynthesis. The widespread use of herbicides has selected for resistance in some common weed species, necessitating the development of novel pest control measures.*

### DECONSTRUCTING THE HERBICIDE-GMO INDUSTRIAL COMPLEX

In the 1960s then U.S. President Dwight Eisenhower warned of the developing military-industrial complex that had formed which resulted in the arms industry influencing military decisions and vice versa. Facetiously, this argument could be applied to a modern view of herbicides and GMO technologies. In some sense, industry wants the user to buy the herbicide resistant crop so that they buy the company’s favorite herbicide. In other words the two technologies are inextricably linked. Though the application of broad-spectrum herbicides in combination with engineered resistant crops has proven commercially successful, this model has led to a lack of innovation (Dayan et al., 2012). A herbicide with a new target site has not been commercialized in nearly 20 years (Dayan et al., 2012). Lack of innovation has resulted in an Ag-chem industry now facing serious challenges, ranging from herbicide-tolerant weed species to the environmental and ecological impacts of herbicide overuse (Benbrook, 2012). The issue of herbicide-resistant weed species has become especially contentious of late, with the emergence of the glyphosate-resistant weed Palmer amaranth now prevalent in 23 states (Gilbert, 2013). From a non-science perspective, public opinion varies widely on the use of GMO-derived food products (Hug, 2008; Bawa and Anilakumar, 2013; Kamle and Ali, 2013), putting further pressure on the Ag-biotech industry. To continue to thrive, the Ag-chem industry should develop innovative new products that circumvent the need for genetic engineering, and are specific to the unmet needs of modern agriculture.

Innovative chemical solutions for crop protection may be informed from studies that predate the GMO era. Most major crop plants are monocots that contend with dicotyledonous weed species, necessitating herbicides that selectively inhibit dicots. This led to the development of broad leaf herbicides that exploit differences between monocot and dicot seedling development. For example, the broad leaf herbicide mesotrione, which inhibits the enzyme 4-hydroxyphenylpyruvate dioxygenase (HPPD), is slowly transported and quickly metabolized by maize (Mitchell et al., 2001). Given that monocots and dicots diverged 140–150 million years ago (Chaw et al., 2004), it is perhaps not surprising that differences in their metabolism can result in a herbicide that is more effective in one class of plants versus another. This divergence does raise questions, however, of whether compounds can be identified that exploit interspecies variation on a smaller scale for agronomic benefit. A first step to addressing these questions is a better understanding of the pharmacogenetic variation across the plant kingdom.

**FIGURE 1:**
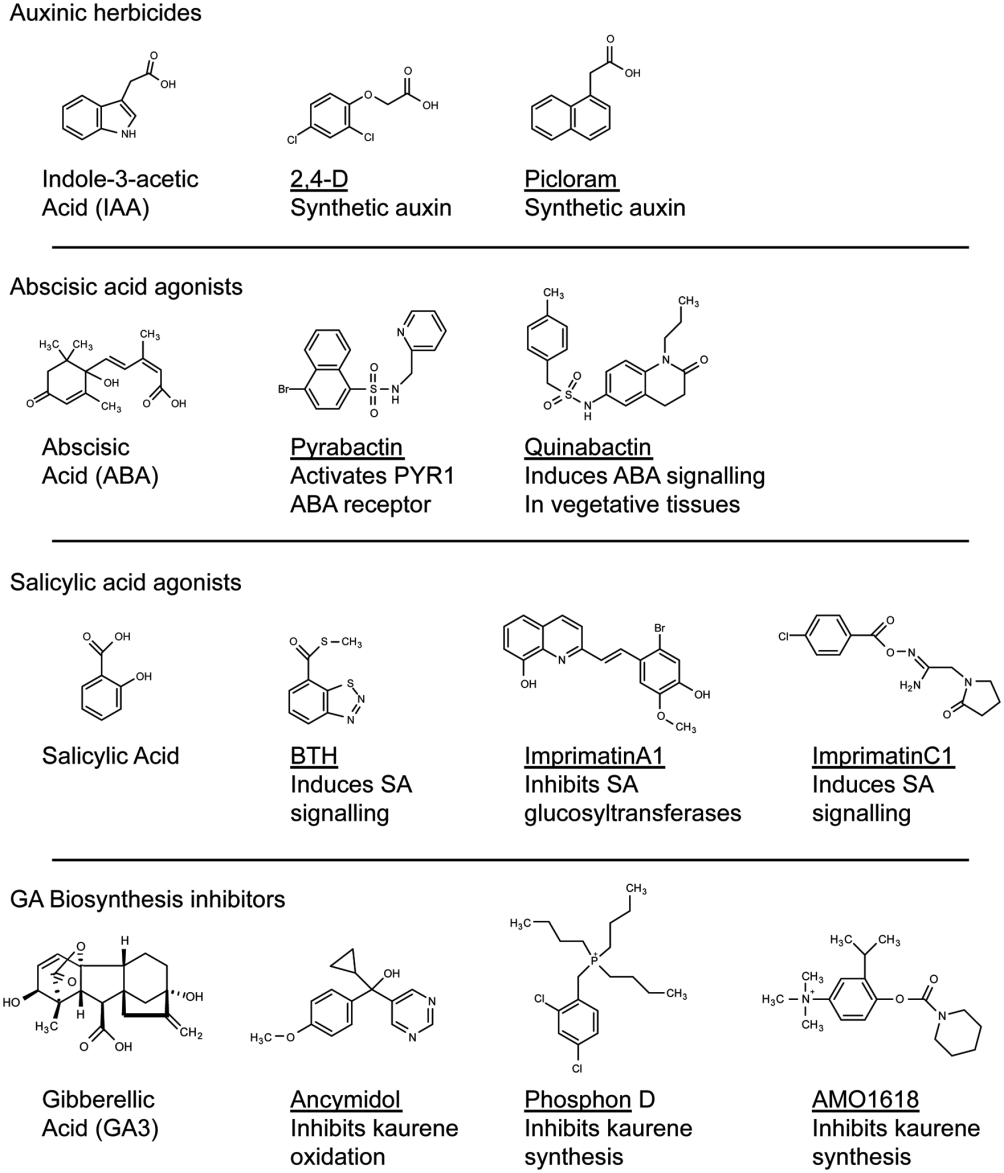
**Structural diversity of compounds that modify plant hormone signaling**. Compounds that modulate hormone signaling have been identified for major plant hormones. By targeting perception or metabolism, these compounds can induce or inhibit hormone activity. High-throughput screening can identify compounds that act on the hormone receptor yet bare no structural resemblance to the natural ligand (for example, compare Pyrabactin with ABA). These compounds can be used as scaffolds in the development of novel agonists or antagonists of plant hormone perception.

The accessibility of modern genomics now affords unparalleled opportunity to query genetic variation across plant species. Genome sequences can be mined to identify species-specific pathways that could form the basis of targeted herbicide treatments. Exploiting interspecific variation that has evolved in essential pathways can enhance herbicide specificity (Walker et al., 1988; Brown, 1990). For example, Auxinic herbicides are thought to mainly target the auxin hormone receptor (Grossmann, 2010). These compounds show species specific potencies based on differences in uptake and metabolism (Sterling and Hall, 1997). Intriguingly, mutants in *Arabidopsis* have been identified that are resistant to the picolinate auxin picloram but not 2,4-D (**Figure 1**; Walsh et al., 2006). One mechanism for this genotype-specific resistance appears to be mutations in one of the five *Arabidopsis* TIR1 auxin receptors (Walsh et al., 2006). Interestingly, a selective resistance to picloram but not to 2,4-D has been documented in the field (Fuerst et al., 1996; Sabba et al., 2003). In principal, these types of studies demonstrate that natural variation in conserved essential pathways could be exploited to develop compounds that inhibit a weed species yet are ineffective in a favored crop. Pharmacogenetic-based bioinformatics could first identify target alleles in weeds and crops that could form the basis of chemical screens for compounds that exhibit specificity toward the weed protein versus the crop version.

### TURNING OVER A NEW LEAF: PROMOTING PRODUCTIVITY, RATHER THAN DEATH

Aside from its role in herbicide discovery, the Ag-biotech industry also has a long history in the development of growth regulators that enhance useful plant attributes (**Figure 1**). From the perspective of plant breeding, genetic manipulation of growth regulators has been central to both horticulture and agriculture. Perhaps the best example involves the impressive yield increases of the green revolution of the 1960s (Davies, 2003), driven by breeding semi-dwarfed varieties for decreased gibberellic acid (GA) biosynthesis in rice and GA signaling in wheat (Hedden, 2003). In parallel to breeding approaches, chemical inhibition of kaurene metabolism, the metabolic precursor to GA, has been used to promote beneficial plant traits in crops (Rademacher, 1991; Gianfagna, 1995). Compounds such as AMO1618 and phosphon D, which inhibit kaurene synthesis, or ancymadol and triazole analogs that inhibit kaurene oxidation, prevent lodging in cereals, increase fruit set in grapes, control size in fruit trees and excessive vegetative growth in cotton (**Figure 1**; Gianfagna, 1995). As an alternative to genetic manipulation and dependence on elite crop varieties, chemical treatments allow farmers the flexibility to adjust crops in response to changing environmental conditions.

These and many other examples of chemical applications for horticultural or agronomic crop improvement were discovered anecdotally by testing known growth regulators on various plant species (**Table A1** in Appendix; Gianfagna, 1995). The chemical genomics era should now allow for a more systematic analysis in this approach. For example, the plant hormone abscisic acid (ABA) has important roles in protecting plants from abiotic stresses such as drought and cold (Ben-Ari, 2012). Rational approaches to this problem have involved attempts to make stable ABA analogs, but have met with limited success (Zaharia et al., 2005). Recently, a chemical screen identified a synthetic naphthalene sulfonamide ABA agonist, Pyrabactin, which preferentially binds an ABA receptor (Park et al., 2009). Although Pyrabactin is mostly active during the germination stage (Park et al., 2009), focused chemical screens built around a sulfonamide substructure identified Quinabactin, which localizes ABA effects to vegetative tissues and results in improved drought tolerance (Okamoto et al., 2013). Thus, stable synthetic compounds that modulates ABA synthesis or signaling may improve the plants response to abiotic stress and promote productivity. It is easy to envision a future in which a farmer that is experiencing drought will apply Quinabactin-like compounds as a treatment to avoid crop losses. By systematically screening for compounds that mimic the activity of a plant hormone, researchers were able to identify chemicals that act through a canonical hormone-signaling pathway. As Pyrabactin bares no structural resemblance to ABA, it is unlikely that modifying the natural ligand to the receptor would have led to the discovery of a compound such as Pyrabactin. This highlights the benefit of screening chemical libraries to identify compounds that can be used as scaffolds in the development of novel agonists or inhibitors of hormone perception.

### BACK TO THE FUTURE

Plant hormones such as auxin, GA, and ABA continue to be excellent targets for the Ag-chemical industry, and have a long-standing history of being manipulated in plant biotechnology (Gianfagna, 1995). Given the successes of these biotechnological advances, other hormones that regulate agriculturally important traits could form the basis of future discovery. Functional analogs of the hormone salicylic acid (SA), such as benzothiadiazole (BTH), promote plant resistance to pathogens and have been developed for use in the field (Gorlach et al., 1996; Lawton et al., 1996). The success of these analogs demonstrated the utility of targeting the SA pathway in the development of compounds that promote crop productivity. This idea formed the basis of high-throughput chemical screens that target SA signaling. Screening through compounds using cell suspension cultures treated to pathogenic *Pseudomonas* identified compounds that promote pathogen resistance in *Arabidopsis* by invoking the hypersensitive cell death pathway in response to pathogen attack (Noutoshi et al., 2012b). The inhibition of SA glucosyltransferases promoted pathogen resistance by increasing SA accumulation (Noutoshi et al., 2012b), whereas a set of functional analogs induced SA signaling *in planta* (Noutoshi et al., 2012a). Whether either of these approaches to plant defense signaling will translate to field applicability remains to be seen, however, this approach has led to viable leads in the development of new agricultural treatments. At the very least, these chemicals can be used to probe SA signaling pathways in plants (Noutoshi et al., 2012a).

Though hormones present an obvious point to manipulate plant output through chemical biology, metabolites can also serve as signaling molecules that impact plant growth and development (Kim et al., 1999; Stevenson et al., 2000; Hirai et al., 2003; Stokes et al., 2013). By definition, proteins involved in metabolism bind small molecules, and should therefore be druggable (Hopkins and Groom, 2002). This should enable the development of chemicals that antagonize metabolic signaling pathways by acting as competitive inhibitors. Over 1/3 of the *Arabidopsis* genome appears to be involved in metabolism, and close to 200,000 enzymes have been annotated across 17 species for which information is available^2^. The plant metabolome may represent an area of untapped potential through which chemical biology can facilitate the development of novel plant growth regulators.

Once a metabolite has been discovered to influence the development of an important plant trait, chemical screens can uncover modifiers of this response. For example, the presence of glutamate influences root system architecture by restricting primary root elongation and promoting the proliferation of lateral roots (Walch-Liu et al., 2006). Modifying root system architecture can benefit plant growth in response to new environments and abiotic stresses (Lynch, 1995; Comas et al., 2013). Screening for compounds that antagonized glutamate perception uncovered novel components of a glutamate signaling pathway and facilitated the development of chemical tools that promote root development in response to endogenous cues (Forde et al., 2013). Targeting metabolic pathways by high-throughput chemical screening should glean new insight into the mechanisms through which metabolites influence plant growth and development.

Directing metabolic output through genetic engineering has been a goal of plant scientists for some time. The development of Golden Rice, engineered to synthesize β-carotene in its seeds (Ye et al., 2000; Paine et al., 2005), demonstrated the potential of metabolic engineering to enhance nutritional value of staple crops (Tang et al., 2009). Vitamin A deficiency is a major health concern in many parts of the developing world, and can result in permanent blindness and death (Underwood and Arthur, 1996). Consuming β-carotene, the precursor to Vitamin A, can help combat malnutrition in some of the world’s poorest populations (Tang et al., 2009). Unfortunately, efforts to implement this technology in regions that stand to benefit the most from it have been stymied by governments and activists in opposition of genetic modification (Enserink, 2008).

Despite the difficulties bringing Golden Rice to the field, it has demonstrated that metabolic engineering can promote nutritional value in crops and raises questions about the ability to use chemicals analogously to genetic engineering in directing the metabolic output of plant. If enzymes make good drug targets then it should be possible to uncover chemicals that can direct metabolic flux by modulating biosynthetic pathways. Presumably, blocking metabolism at crucial time-points during plant development can promote the accumulation of specific metabolites that could have economic or nutritional value. Methods that enable quick assessment of metabolite abundance would facilitate screens in search of compounds that promote the accumulation of metabolites of interest. Though we are not necessarily advocating for increased application of chemicals to food products, we believe that targeted manipulation of plant metabolism through chemical biology does have the potential to promote nutritional value in crops and to enhance the accumulation of rare or expensive natural products in some species.

Similarity between crop species is beneficial to plant researchers because treatments that are effective in one species are likely to be useful in a related species. In principal, this should facilitate translation from laboratory science to real-world applications. Despite this, there seems to be a paucity of published examples in which leads from high-throughput screens in *Arabidopsis* were then tested across agriculturally important species. Some characteristics of the model plant *Arabidopsis*, including its small size and rapid growth, make it an obvious choice as the subject of phenotype-based high-throughput chemical screening (Robert et al., 2009). As sequenced genomes become readily available and as new tools are developed for other plant species, compounds identified using *Arabidopsis* should be assayed in other plants to assess the utility of these leads in commercial applications. Focus on the development of compounds that modify traits in important species might encourage collaboration between the Ag-biotech sector and academic research groups, a relationship that has stimulated innovation in the pharmaceutical industry (Scudellari, 2011; Loregian and Palu, 2013).

### LESSONS FROM BIG PHARMA

In many ways, the Ag-chem industry is facing a similar situation to the pharmaceutical industry, in which exorbitant costs of drug development have become prohibitive. This has resulted in a stagnating supply of innovative new products coming through the research and development pipeline (Bennani, 2011; Pammolli et al., 2011). The increasing market share being lost to generics and some valuable patents expiring over the past few years have put pressure on Big Pharma to restructure their lead development strategy (Cuatrecasas, 2006; Loregian and Palu, 2013). Over the past decade, an increasing number of large pharmaceutical companies have established fruitful collaboration with academic research laboratories, effectively “outsourcing” discovery-based lead generation (Scudellari, 2011; Fishburn, 2013). In support of this, universities across the world have established high-throughput screening facilities that enable drug discovery (Loregian and Palu, 2013). In this model, discovery-based research is handled by the academic institution; commercialization and product development are generally managed by the corporation.

This relationship has allowed the burden of high-risk projects to be taken by the research institute, whose incentives and measures of success may differ from that of the corporate partner (Fishburn, 2013; Loregian and Palu, 2013). An academic group may put greater value in publications and training opportunities (Loregian and Palu, 2013), or may be more interested in pursuing high-risk projects that attempt to drug difficult targets, such as transcription factors and protein–protein interactions (Loregian and Palu, 2013). In this sense, the needs of society benefit from close collaboration between academic labs and Big Pharma. These collaborations can mean more attention paid to rare or neglected diseases, greater propensity to tackle historically difficult targets, and the generation of new molecular entities that can be developed into therapeutic treatments.

A similar strategy would benefit the Ag-biotech industry, in which academic chemical biology labs could make use of the available high-content screening platforms to develop new herbicides and agricultural chemicals. The prevalence of herbicide-resistant weeds, coupled with the increased abiotic stresses crippling agricultural output are putting pressure on the Ag-chem industry to develop innovative methods of crop protection that sidestep the need for genetic modification. Modern genomic analysis should enable researchers to quickly understand the mechanism of resistance, and scientists now have the tools available to develop tailored chemical treatments that target specific classes of weeds and other pests. Taking a lead from Big Pharma, the private sector and academic laboratories should collaborate to establish translational research programs that promote innovation and open new opportunities to sustain agricultural productivity.

## Conflict of Interest Statement

The authors declare that the research was conducted in the absence of any commercial or financial relationships that could be construed as a potential conflict of interest.

## Footnotes

1 http://www.genet.sickkids.on.ca/cftr

2 http://www.plantcyc.org

## APPENDIX

**Table A1:** Plant growth regulators used in agriculture.

Growth regulator	Mode of action	Agronomic benefit	Species
**Hormone action**			
IBA	Auxin analog	Rooting of stem cuttings	Many plants
2,4-D	Auxin analog	Prevent fruit drop	Apple, pear, citrus
4-CPA	Auxin analog	Stimulate fruit setting	Tomato
NAA	Auxin analog	Fruit thinning	Apples, pear
GA	Plant hormone	Fruit size increase	Grapes
GA	Plant hormone	Delay fruit ripening	Apple, pear, citrus
GA	Plant hormone	Increase yield	Sugarcane
GA	Release enzymes	Malting	Barley
Ethephon	Ethylene release	Rubber production	Hevea
Ethephon	Ethylene release	Promote abscission	Cherry, walnut, olive
Ethephon	Ethylene release	Fruit ripening	Apple, tomato
Ethephon	Ethylene release	Color, fruit acidity	Grapes
Ethephon	Ethylene release	Promoting leaf senescence	Tobacco
Benzyladenine (BA)	Cytokinin analog	Lateral bud formation	White pine
Accel	Cytokinin analog	Increase lateral branching	Carnations
Promalin	Mixture GA_4/7_+BA	Fruit diameter	Apple
Promalin	Mixture GA_4/7_+BA	Increase lateral branching	Apple trees
**Growth retardants**			
Chloromequat	GA synthesis inhibitor	Stem growth	Poinsettia
Ancymidol	GA synthesis inhibitor	Stem growth	Easter lily
Mepiquat	GA synthesis inhibitor	Reduce excessive growth	Cotton
Chlorflurenol	GA synthesis inhibitor	Reduce growth	Turf grass
Diaminozide	GA synthesis inhibitor	Increase fruit set	Grapes
Diaminozide	GA synthesis inhibitor	Color	Cherry
Diaminozide	GA synthesis inhibitor	Flower bud formation	Apple, pear
Paclobutrazol	GA synthesis inhibitor	Control tree size	Fruit trees
**Miscellaneous**			
Maleic hydrazide	Unknown	Inhibit sprouting	Onion, potato
Glyphosine	Glyphosate analog	Increases sugar yield	Sugarcane
Dimethipin	Unknown	Defoliant	Cotton

*Chemicals that promote a variety of agriculturally important growth traits have been developed for use in the field.*
